# Highly Stable Glassy Carbon Interfaces for Long-Term Neural Stimulation and Low-Noise Recording of Brain Activity

**DOI:** 10.1038/srep40332

**Published:** 2017-01-13

**Authors:** Maria Vomero, Elisa Castagnola, Francesca Ciarpella, Emma Maggiolini, Noah Goshi, Elena Zucchini, Stefano Carli, Luciano Fadiga, Sam Kassegne, Davide Ricci

**Affiliations:** 1 MEMS Research Lab., Department of Mechanical Engineering, College of Engineering, San Diego State University, 5500 Campanile Drive, San Diego, CA 92182-1323, USA; 2 Center for Sensorimotor Neural Engineering (CSNE), Box 37, 1414 NE 42nd St., Suite 204, Seattle, WA 98105-6271, USA; 3 Center for Translational Neurophysiology of Speech and Communication, Istituto Italiano di Tecnologia, Via Fossato di Mortara 17/19, 44121, Ferrara, Italy; 4 Section of Human Physiology, University of Ferrara, Via Fossato di Mortara 17/19, 44121, Ferrara, Italy

## Abstract

We report on the superior electrochemical properties, *in-vivo* performance and long term stability under electrical stimulation of a new electrode material fabricated from lithographically patterned glassy carbon. For a direct comparison with conventional metal electrodes, similar ultra-flexible, micro-electrocorticography (μ-ECoG) arrays with platinum (Pt) or glassy carbon (GC) electrodes were manufactured. The GC microelectrodes have more than 70% wider electrochemical window and 70% higher CTC (charge transfer capacity) than Pt microelectrodes of similar geometry. Moreover, we demonstrate that the GC microelectrodes can withstand at least 5 million pulses at 0.45 mC/cm^2^ charge density with less than 7.5% impedance change, while the Pt microelectrodes delaminated after 1 million pulses. Additionally, poly(3,4-ethylenedioxythiophene)-poly(styrenesulfonate) (PEDOT-PSS) was selectively electrodeposited on both sets of devices to specifically reduce their impedances for smaller diameters (<60 μm). We observed that PEDOT-PSS adhered significantly better to GC than Pt, and allowed drastic reduction of electrode size while maintaining same amount of delivered current. The electrode arrays biocompatibility was demonstrated through *in-vitro* cell viability experiments, while acute *in vivo* characterization was performed in rats and showed that GC microelectrode arrays recorded somatosensory evoked potentials (SEP) with an almost twice SNR (signal-to-noise ratio) when compared to the Pt ones.

Neural implants have been used for decades to uncover fundamental knowledge in the workings of the nervous system, treat several neurological disorders and, more recently, to connect neural prosthetics for restoring lost sensorimotor functions^1^,^2^,^3^,^4^. As a rule, a major goal of neural implant research is to integrate therapeutic devices into the nervous system without triggering a severe tissue reaction^5^. As the field of neurophysiology has become more mature and sophisticated, the need of high-resolution neural implants has also become pressing. Specifically, micro-ECoG array technology has seen a decrease of the electrodes size - which improves their spatial selectivity - and a reduction of the inter-electrode distance - which increases the spatial resolution of the devices^2^,^6^,^7^,^8^,^9^,^10^,^11^. Although it potentially helps with signal resolution, the reduction in the electrodes size also has the negative effect of raising the impedance values of most traditional electrode materials outside the ideal range, leading to larger thermal noise levels and, consequently, to lower signal- to-noise ratio^1^,^12^. Additionally, for all implantable neural devices, the electrochemical properties and long-term stability of the electrode material are of particular importance, as any deterioration of the electrode or induction of inflammatory reactions at the neural interface can potentially harm the surrounding tissue^1^,^13^,^14^. Thus, significant research has so far been dedicated to improving traditional metallic neural interfaces through the integration of new materials onto implanted microelectrode arrays (MEAs) that can reduce the long-term tissue response to the implanted device^15^,^16^,^17^,^18^,^19^,^20^,^21^,^22^,^23^,^24^. These new materials offer several advantages, including: lower electrochemical impedance (which improves the signal-to-noise ratio (SNR) and allows for miniaturization), wider electrochemical windows that prevent faradaic reactions, and higher charge injection capabilities that enable more efficient electrical stimulation of the nervous tissue^15^,^16^,^17^,^18^,^19^,^20^,^21^,^22^,^23^,^24^.

Glassy carbon (GC) is a very attractive material for use in neural interfaces as it has been demonstrated to have good electrical properties, to be chemically inert and electrochemically stable, and amenable to being fabricated into a variety of geometries^25^,^26^,^27^,^28^,^29^,^30^,^31^. However, only recently has GC been considered for such a use, mainly due to new enabling technologies that allow for pattern transfer and the integration of GC electrodes into thin-film polymeric substrates^19^,^20^,^21^. This achievement could exert a great impact in the field of neural prosthetics, as devices fabricated from thin-film, ultra-flexible polymer substrates reduce the mechanical mismatch between the device and tissue, lessening the inflammatory reactive response. In addition, ultra-flexible polymers have been shown to delay contact encapsulation - and therefore to increase the lifespan - of neural prostheses^32^,^33^,^34^. Moreover, it has been reported that polyimide (a commonly used thin-film substrate) has better adhesion to carbon based materials, as compared to traditionally used noble metals^19^.

In this work, we report on a new class of neural prostheses, fabricated with GC electrodes housed in an ultra-flexible, thin-film polyimide substrate that is able to leverage the superior electrochemical stability of glassy carbon along with the improved biocompatibility of thin-film devices. We compare, for the first time, biocompatibility, electrochemical properties and *in vivo* performance of thin-film MEAs fabricated with either GC or Pt, a biocompatible material traditionally used for neural interfaces, with particular focus on the long term stability. Furthermore, we compare GC and Pt as substrates for Poly(3,4-ethylenedioxythiophene)-poly(styrenesulfonate) (PEDOT-PSS), a highly conductive polymer with great chemical stability that is often used^16^,^18^,^22^,^23^,^35^,^36^,^37^,^38^,^39^,^40^ to improve electrochemical performances when electrode miniaturization^41^ and high-density spatial arrays are required. We believe that these wide spectra of *in vitro* and *in vivo* results demonstrate that GC electrodes offer a new and compelling material for neural recording and stimulation.

## Results and Discussion

### Device Fabrication

**Figure 1:**
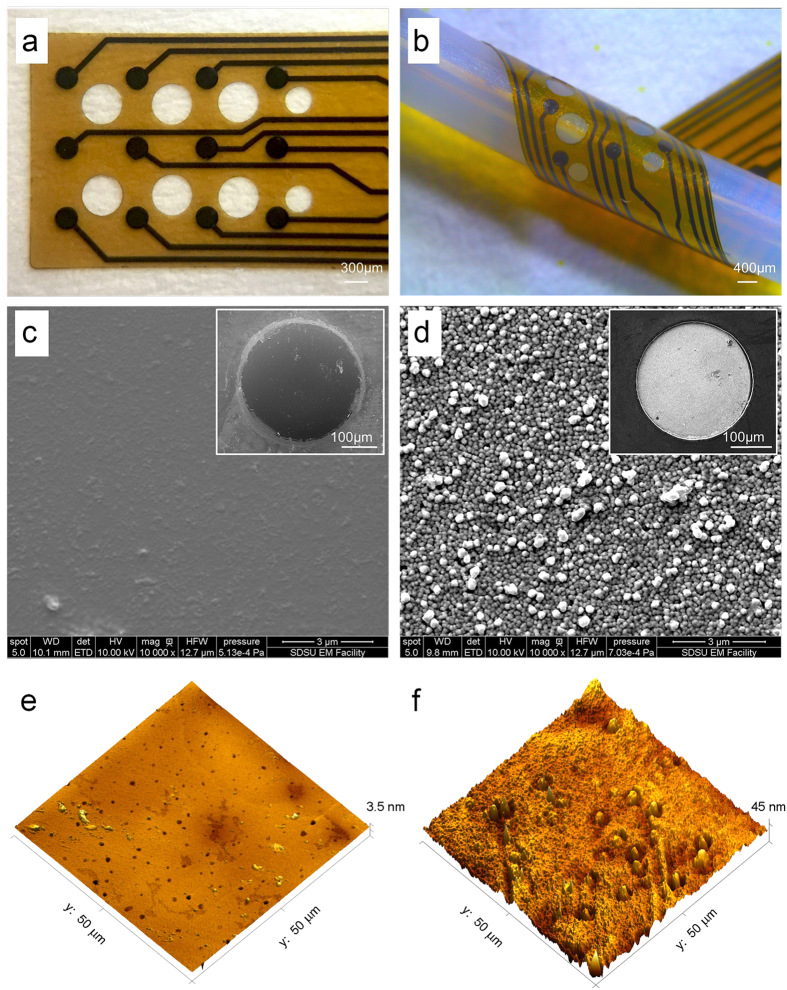
Representative images of GC and Pt thin-film electrode arrays. GC 12 electrode array (**a**). In (**b**) the device is folded to show its flexibility. Scanning electron microscopy (SEM) images of the different electrode materials: (**c**) GC and (**d**) Pt. Both are taken at same magnification (10000×). (**e**) AFM image of GC electrode. (**f**) AFM of Pt electrode showing rough surface with mean roughness of 35.0 nm.

In order to characterize neural recording and stimulating capabilities of GC microelectrodes integrated into thin-film ultra-flexible arrays, we performed a variety of *in vitro* and *in vivo* tests and compared the results with those obtained using a traditional metallic neural interface (Pt). Newly introduced pattern transfer techniques^20^ were used to fabricate GC based ultra-flexible, thin-film microelectrode arrays (MEAs) for ECoG applications with a total thickness of approximately 15 μm. Standard metal lift-off processes were used to fabricate a similar MEA containing Pt electrodes with the same geometry and thickness. The array layout was tailored to the neurophysiological experiment conducted for this study, with each device containing an array of 12 electrodes (diameter 300 μm) with a pitch of 1 mm. Optical images of the whole device along with SEM and AFM images of the different electrode materials are shown in Fig. 1. Morphology analysis of AFM images of Pt electrodes indicate mean roughness of 35.0 nm while that of GC electrodes show mean roughness of 3.3 nm (n = 5), an order of magnitude difference.

### Electrochemical characterization

**Figure 2:**
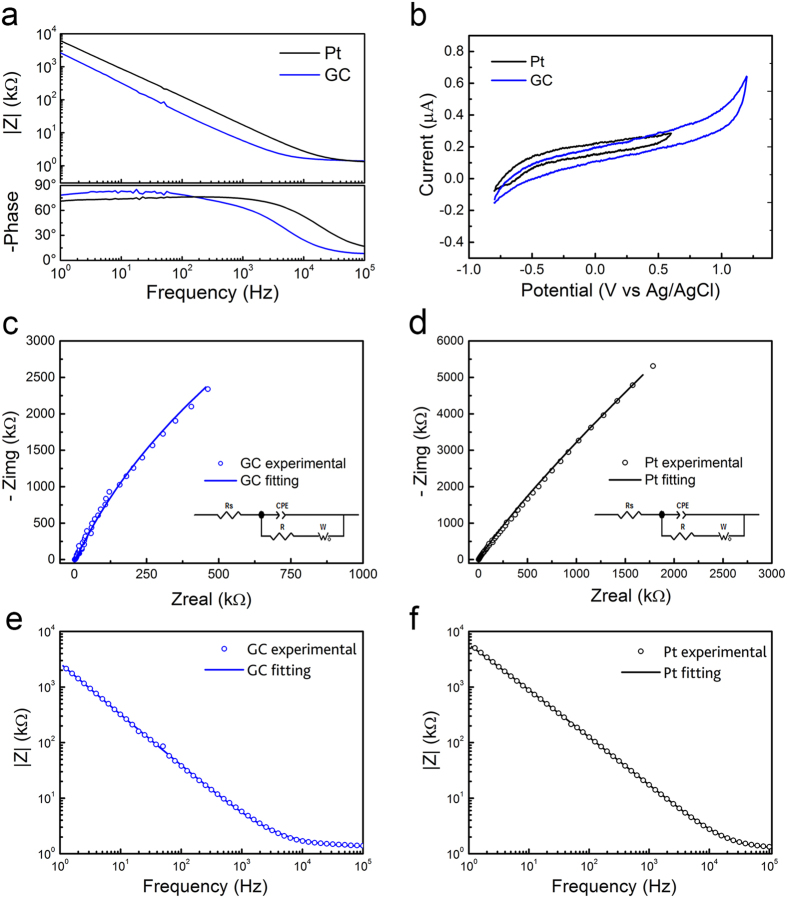
Comparison between GC (blue) and Pt (black) 300 μm Ø electrodes. (**a**) EIS; (**b**) CV plots; (**c**) and (**d**) impedance Nyquist plots of experimental data and equivalent circuit models; (**e**) and (**f**) Bode plots of the impedance modulus of experimental data and equivalent circuit models. See text for abbreviations.

The electrochemical behavior of GC and Pt microelectrodes was studied by means of linear sweep voltammetry (LSV) — to define the electrochemical window (EW) — cyclic voltammetry (CV) — to quantify their capacitive charging — and electrochemical impedance spectroscopy (EIS) — to give insights of charge transport dynamics. GC microelectrodes exhibited lower impedance than their Pt counterparts across the whole frequency range, as shown in the module of the Bode plot (Fig. 2a). The impedance values respectively at 10 Hz, 100 Hz and 1 kHz (mean and standard deviation, n = 10) for both Pt and GC microelectrodes are reported in Supplementary Table S1. LSV tests proved that the EW of GC is significantly wider than that of Pt (2.4 V vs. 1.4 V, see Fig. 2b and Supplementary Fig. S1), confirming data reported in literature for both materials^1^,^42^. In addition, the voltammograms (Supplementary Fig. S1) are featureless within the EW, indicating that no faradic reactions occur and that current is delivered through the charging and discharging of the interfacial double layer. This capacitive charge injection mechanism is ideal for neural stimulation because no chemical changes occur at the surface of the electrode in contact with the tissue. Thus, these findings suggest that GC is an appropriate material for ECoG recordings. As expected, CV studies confirmed the wider EW of GC and that the charge transfer capacity (CTC), calculated as the time-integral of an entire CV cycle between the EW limits, is also higher for GC (14.3 ± 3.8 vs. 8.6 ± 3.5 mC/cm^2^, see Supplementary Table S1). CV and LSV results imply that GC electrodes have wider operating range than Pt electrodes under stimulation, as they are able to withstand greater voltage excursions without producing irreversible faradaic reactions.

Equivalent circuit models were used to analyze experimental EIS data providing further insight into the charge transfer properties of the electrodes. For both GC and Pt microelectrodes, the optimal circuit model was composed by the electrolyte resistance (R_S_), a charge transfer resistance (R_CT_) in parallel with a constant phase element (CPE)^43^ and the Warburg diffusion impedance (W_S_)^44^, as shown in the inset of Fig. 2c,d. Pt-based electrodes are known to exhibit faradaic/capacitive mechanisms that strongly influence the nature of the impedance plot, especially at low frequencies^1^. For the GC-based electrodes, such behavior may be ascribed to the formation of electroactive groups (for example quinoidal structures, and/or carbonyl and hydroxyl groups^30^) or non-conductive species during its preparation process based on the pyrolytic decomposition of a precursor polymer. This pseudo-faradaic behavior was also reported by Heiduschka *et al*.^45^ for activated GC in aqueous KCl based electrolyte. In Table 1 we report the fitted values for representative Pt and GC electrodes: EIS fitting confirmed the higher capacity of GC vs. Pt, in terms of the Q_o_ value representing the capacitance at ω = 1 rad s^−1^, in accordance with CV characterization (see Supplementary Table S1 for CTC). It can be also observed that the Warburg impedance^46^ significantly contributes in lowering the total impedance of GC. In particular, the Warburg admittance Y_0_ is three orders of magnitude higher in the case of GC and this may suggest a better diffusion of the electrolyte within the GC electroactive surface^44^.

### GC Stability

The ability of GC and Pt microelectrodes to resist intense and prolonged current stimulation patterns was verified by repeatedly applying a series of cathodic-first charge balanced biphasic current pulses with 1 mA current amplitude, 300 μs cathodic half-phase period and 1 ms period in saline solution (0.9% NaCl). This corresponded to a cathodic charge density of 0.43 mC/cm^2^, which is within the range of the charge injection limit - defined as the maximum quantity of charge an electrode can inject before reaching the water electrolysis potential - previously calculated for metal microelectrodes^35^, and comparable to the one used to evoke sensations in human using millimeter-scale surface ECoG electrodes^47^.

**Figure 3:**
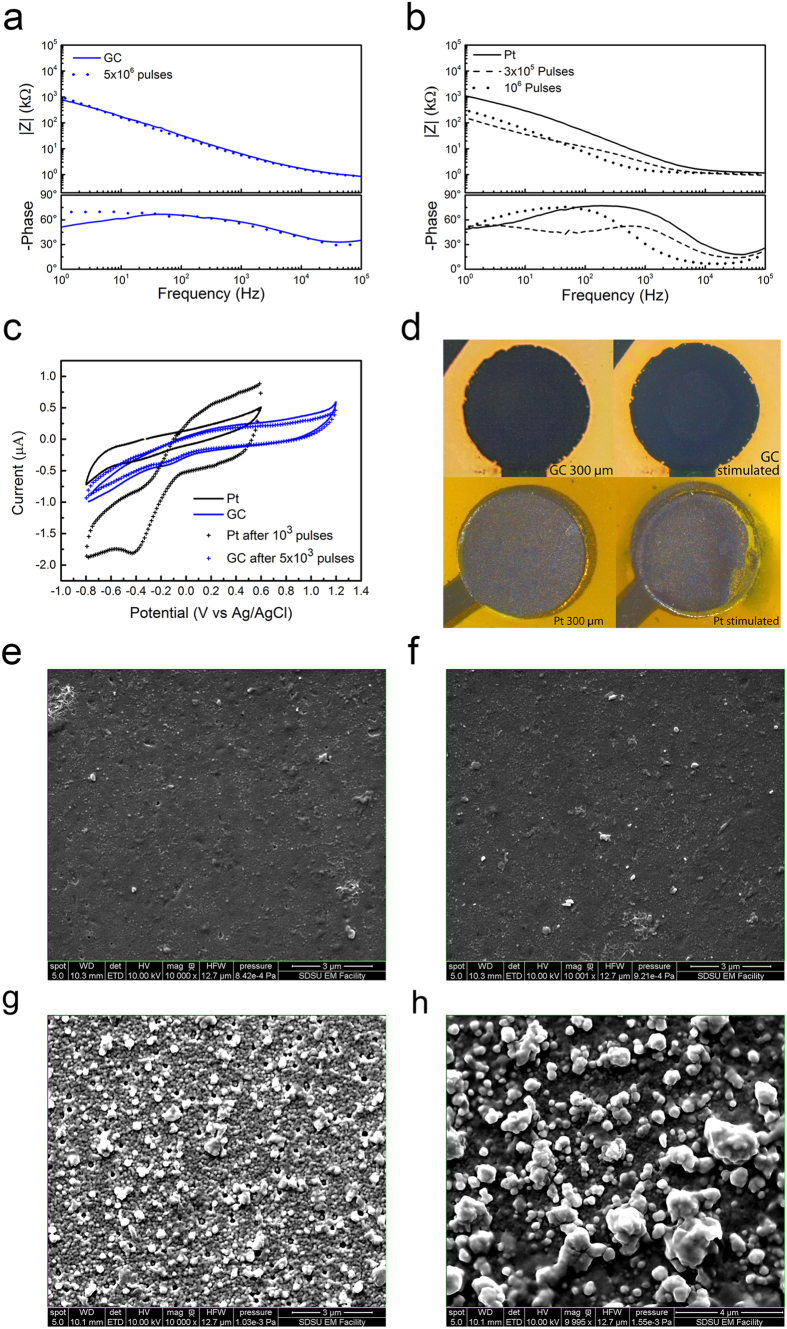
Comparison of electrode properties before and after stimulation experiments. Representative impedance spectra before and after stimulation experiments of GC (**a**) and Pt (**b**) microelectrodes. (**c**) Representative cyclic voltammograms of Pt (black) and GC (blue), before and after stimulation experiments. (**d**) Representative Optical pictures of the overall 300 μm Ø Pt and GC electrodes before and after 1 and 5.0E6 pulses (respectively **e**,**f**,**g** and **h**). Representative SEM images before and after stimulation; GC electrode before (**e**) and after (**f**) 5.0E6 pulses. Pt electrode before (**g**) and after (**h**) 1.0E6 pulses. See text for abbreviations.

GC electrodes were able to withstand 5.0E6 biphasic pulses with negligible change in EIS (Fig. 3a) and CV (Fig. 3c), while Pt electrodes showed significant changes to both the EIS and CV plots after 1.0E6 pulses (Fig. 3b,c). SEM and optical microscopy validated these results with the Pt electrodes undergoing visible corrosion after the 1.0E6 pulses while the GC electrodes remaining unchanged. In particular, after 3.0E5 and 1.0E6 pulses, there is a significant drop in impedance of the Pt electrodes throughout the frequency range, as can be observed in Fig. 3b (dotted line). This reduction in the impedance modulus is due to the chemical modification of the surface of the electrode. In fact, the CV plot of Pt under stimulation (Fig. 3c) suggests the dissolution of the Pt surface and shows the appearance of a new redox process, which is presumably related to the Cr(II)/Cr(III) redox couple (E° = −0.42 V vs. NHE)^48^ on the exposed chromium underlayer. Pt delamination after 1.0E6 pulses is also clearly shown by optical images (Fig. 3d) indicating an important detachment of Pt from the electrode substrate, whereas GC based electrodes remained identical over the entire duration of the test.

The corrosion of the Pt electrodes - due to the applied electrical pulses - made visible the underlying layer of Cr, used as adhesion promoter during the MEMS fabrication process. The change in color of the Pt electrode shown in Fig. 3d, in fact, can be attributed to Cr(III) oxide traces on the surface of the corroded metal electrode. Finally, SEM image analysis corroborates this result: while the morphology of GC electrodes remained essentially unchanged after 5.0E6 pulses (Fig. 3e,f), the roughness of Pt electrodes increased after 1.0E6 pulses (Fig. 3g,h), further confirming the instability of Pt under these stimulation conditions.

### Electrodeposited PEDOT-PSS on GC and Pt substrates

**Figure 4:**
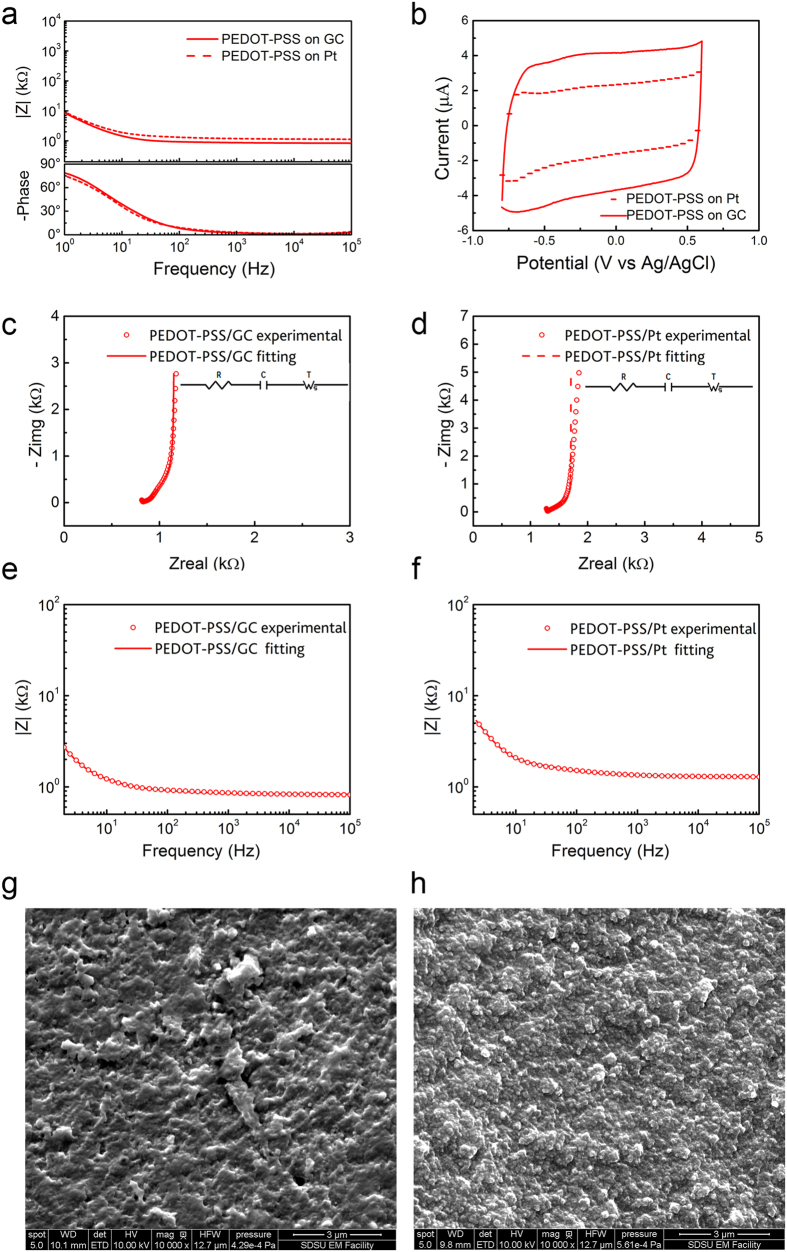
Comparison between PEDOT-PSS coating on GC and Pt 300 μm Ø electrodes. (**a**) EIS; (**b**) CV plots; (**c**) and (**d**) impedance Nyquist plots of experimental data and equivalent circuit models; (**e**) and (**f**) Bode plots of the impedance modulus of experimental data and equivalent circuit models. Representative SEM images of PEDOT-PSS morphology on GC (**g**) and Pt (**h**) substrates.

To improve the electrochemical properties of the conductive surface, aiming to further miniaturize the electrodes^41^, we electrodeposited PEDOT-PSS on GC and Pt and studied the ability of the conductive polymer to grow and adhere to the two different substrates. PEDOT-PSS can be electropolymerized by means of potentiodynamic (PD, cycling potential), potentiostatic (PS, constant potential) or galvanostatic (GS, constant current) electrochemical routes. It has been reported by Cui *et al*. that PEDOT-PSS deposited on Pt microelectrodes by galvanostatic mode is prone to delaminate, depending on the thickness of the polymer film^49^. In particular they observed that this physical degradation is more pronounced as the thickness increases. Among the methods we have tested, we were able to obtain homogeneous polymer films on our GC electrodes by the PD method, that has revealed to be the optimal choice in terms of reproducibility and morphology. These findings are in accordance with the recently published study by Castagnola V. and co-workers^50^ reporting how PD, with respect to PS and GS, leads to a more uniform film electropolymerization that finally produced the most homogeneous, smooth deposition and high stability. They also reported that PS and GS routes are more impacted by the quality of the electrode pristine surface. As expected, after PEDOT-PSS electrodeposition, the CTC of both Pt and GC electrodes significantly increased (Fig. 4b, Supplementary Table S1) and their impedance decreased over the entire frequency range (1–10^5^ Hz), especially between 1 and 100 Hz, where ECoG signals express their maximum power. Such behavior can be attributed to a significant increase in the electrodes surface area consequent to the adsorption of PEDOT-PSS onto the Pt and GC substrates, and to the electrical conduction mechanism of PEDOT-PSS, that transfers both ionic and electronic current, enhancing the efficiency of signal transduction^36^,^51^,^52^. However, the CTC of the PEDOT-PSS-coated GC electrodes was nearly double the CTC of the PEDOT-PSS-coated Pt ones (Fig. 4b, Supplementary Table S1). This suggests that the nature of the underlayer (i.e. GC or Pt) strongly affects polymer growth and, as a consequence, the properties of the deposited PEDOT-PSS film. Representative SEM images of PEDOT-PSS coating on GC and Pt electrode substrates are reported in Fig. 4g,h.

After depositing PEDOT-PSS onto Pt and GC electrodes, the EIS plots are entirely dominated by the electrochemical properties of the conductive polymer. The Nyquist plots show the typical 90° capacitive line in the low frequency region (see Fig. 4c,d), whereas diffusion dominates the high frequency domain, as indicated by the characteristic 45° line. Thus, the circuit model *RCT,* where R represents the electrolyte resistance, C the capacitance and T the finite-length Warburg diffusion impedance, (as shown in the inset of Fig. 4c,d), was used to fit EIS data for PEDOT based electrodes^53^. The lower impedance observed for PEDOT-PSS coatings on GC electrodes can be ascribed to a reduced diffusional impedance R_D_, together with a higher total capacitance C_EIS_ due to a higher active area, as summarized in Table 2. Moreover, we found a good correlation between capacitance obtained by the fitting and the capacitance deduced by cyclic voltammetry (C_CV_ = *I*/*v* where *v* is the scan rate, and *I* is the average of the cathodic and anodic currents, see Fig. 4b and Table 2), corroborating the optimal circuit model *RCT*.

The stability of PEDOT-PSS coatings onto GC and Pt substrates was investigated using the same stimulation protocol outlined for the 300 μm Ø GC and Pt electrodes. The PEDOT-PSS-coated GC electrodes were able to withstand 5.0E6 biphasic pulses without any reduction of the redox activity, as confirmed by the negligible changes in both EIS and CV plots (see Supplementary Fig. S3). On the contrary, for PEDOT-PSS-coated Pt electrodes the EIS plot shows a progressive increase of the impedance modulus and the appearance of a new faradaic process, with a time constant in the order of 16 ms indicating the delamination or degradation of the conductive polymer layer. This is also confirmed by CV plots obtained after 1.0E6 pulses that show a reduction in the capacitance and a progressive downshift of the redox potential. Finally, SEM images validated these results, with the PEDOT-PSS coating on Pt electrode beginning to delaminate and form small fractures after 1.0E6 pulses, while the morphology of PEDOT-PSS on GC electrode can sustain 5.0E6 pulses without evident cracks due to delamination (Supplementary Fig. S3).

### GC and PEDOT-PSS combination for electrodes miniaturization

**Figure 5:**
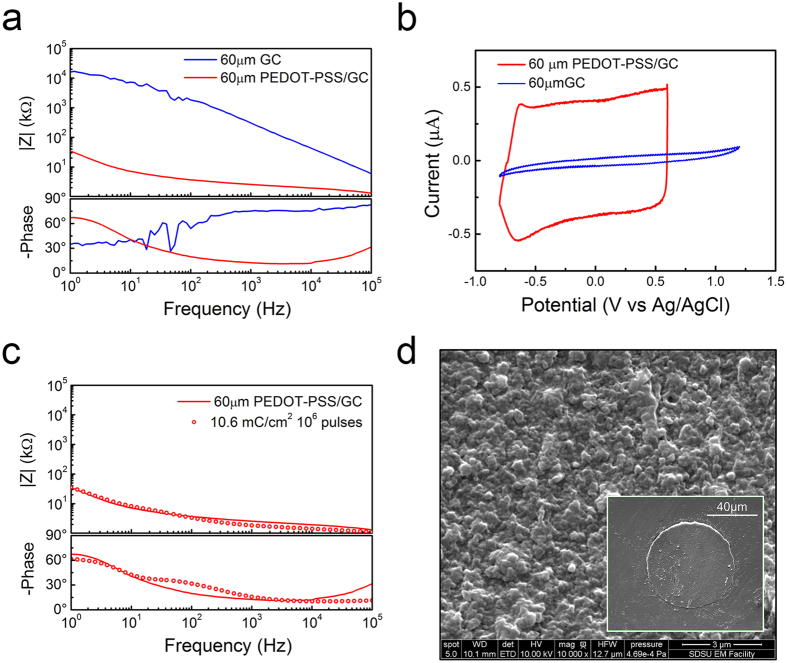
Effect of PEDOT-PSS coating on miniaturized electrodes. Representative impedance spectra (**a**) and voltammograms (**b**) of a 60 μm Ø GC microelectrodes before and after PEDOT-PSS coating. (**c**) Impedance spectra of PEDOT-PSS coating on GC before and after stimulation experiments. (**d**) Representative SEM images of PEDOT-PSS coating on 60 μm Ø GC microelectrodes after 1.0E6 pulses.

Having demonstrated that PEDOT-PSS modified GC microelectrodes exhibit both superior electrochemical proprieties and higher stimulation capability, we studied PEDOT-PSS coatings on miniaturized GC microelectrode. As expected, we confirm the ability for PEDOT-PSS coatings to dramatically decrease the impedance of miniaturized GC electrodes through the electrodeposition of PEDOT-PSS on 60 μm diameter GC electrodes. Representative impedance spectra and voltammograms of 60 μm GC microelectrodes before and after PEDOT-PSS electrodeposition are reported in Fig. 5. The effects of the PEDOT-PSS coating are even more evident in the case of small electrodes, which display a significant decrease of the impedance over the entire frequency range (1–10^5^ Hz), and in particular, an impedance decrease of more than three orders of magnitude in the 10–100 Hz frequency band, from 1.1E4 ± 6.4E3 kΩ to 12.4 ± 2.4 kΩ at 10 Hz (Fig. 5a, Supplementary Table S1). We observed absolute impedance values at 1 kHz that are in a similar range to the values of the 300 μm Ø PEDOT-PSS coated electrodes (0.9 ± 0.1 kΩ versus 3.6 ± 0.4 kΩ at 1 kHz) and, not surprisingly, the CTC of the 60 μm Ø PEDOT-PSS coated electrodes drastically increases from 46.9 ± 8.9 to 893.5 ± 137.8 mC/cm^2^. The enhanced CTC, combined with their low impedance, is an essential feature that opens the possibility to safely use these miniaturized electrodes for stimulation while maintaining excellent recording performance. To prove this assumption, we applied to the 60 μm PEDOT-PSS coated electrodes the same stimulation pattern used for the 300 μm Ø electrodes. In this case, the current of 1 mA for 300 μs cathodic half-phase period corresponded to an extremely high cathodic charge density of ~10 mC/cm^2^. We noted that the miniaturized microelectrodes were able to withstand 1.0E6 biphasic pulses with negligible change in EIS (Fig. 5) and maintained a compact PEDOT-PSS coating without evident delamination (Fig. 5d). This, therefore, demonstrates the ability to inject current sufficient enough to evoke sensation in humans using small microelectrodes, thanks to the combination of a stable GC electrode and high surface area PEDOT-PSS coatings. This aspect is of fundamental importance for micro-ECoG array technologies, because miniaturized low-impedance recording sites not only allow improved sensitivity and spatial selectivity (thereby increasing the information content of the recorded signal) but also allow reduction of inter-electrode distance (pitch), enhancing their spatial resolution^22^.

### Acute *in -vivo* recordings

**Figure 6:**
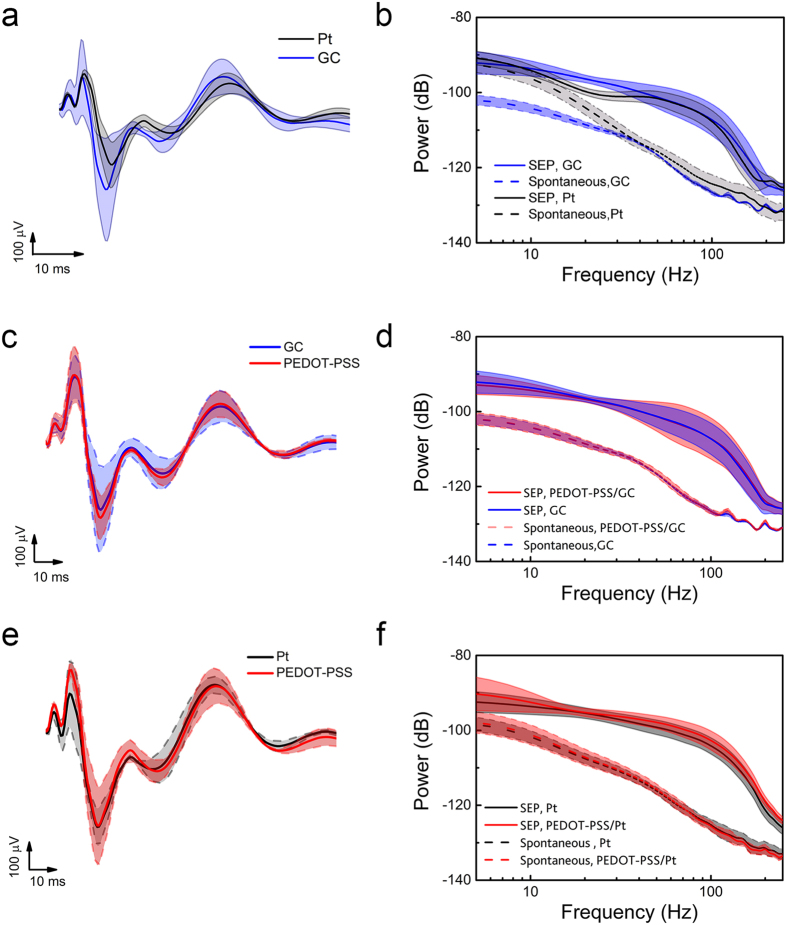
Electrode *in-vivo* recording performance. Averaged SEPs elicited in the vibrissa representation of S1 by electrical stimulation of trigeminal nerves recorded from each of the 12 channels. (**a**) Traces obtained by averaging the SEP evoked by 100 stimulation patterns for each 300 μm Ø Pt (black) and 300 μm Ø GC (blue) microelectrodes. (**b**) Corresponding spectral power densities (SPDs) obtained for the non-filtered SEP activity and the spontaneous activity recorded from rat somatosensory cortex using Pt (black) and GC (blue) microelectrodes. (**c**) Averaged SEPs evoked by 100 stimuli for each GC (blue) and GC-PEDOT-PSS (red) microelectrode. (**d**) Corresponding SPDs obtained for the non-filtered SEP activity and the spontaneous activity recorded from the rat brain using 300 μm Ø GC (blue) and GC-PEDOT-PSS (red) microelectrodes. (**e**) Traces obtained by averaging the SEPs evoked by 100 stimulation patterns for each 300 μm Ø Pt (black) and 300 μm Ø Pt-PEDOT-PSS (red) microelectrodes. (**f**) Corresponding SPDs obtained for the non-filtered SEP activity and the spontaneous activity recorded from the rat brain using Pt (black) and Pt-PEDOT-PSS (red) microelectrodes.

To validate the recording capability of the micro-ECoG arrays, we tested the devices *in vivo* by recording neural signals (somatosensory evoked potentials, SEPs) from eight different locations of rat somatosensory cortex (S1). Each MEA (2.1 × 3.1 mm) hosted 12 micro-recording sites with 1 mm pitch. Typical SEPs elicited in the S1 by the electrical stimulation of trigeminal nerve are shown in Fig. 6a,c and e. Mean traces were obtained by averaging the SEPs evoked by 100 stimulation patterns for each GC (blue), Pt (black) and PEDOT-PSS (red) coated microelectrodes. The quality of the recorded neural signals was evaluated in terms of signal power and signal-to-noise ratio (SNR). The estimated spectral power densities (SPDs) of the spontaneous and evoked neural activities recorded from the rat cortex were characterized as described in the Methods section, while the SNR was calculated as the ratio between the signal power (integral of the signal SPD) and the integral of the SPD of the noise (average of 10 min of spontaneous neural activity recording from eight different positions). The SNR values (low frequency band 0–250 Hz) of Pt, GC, PEDOT-PSS coated Pt and PEDOT-PSS coated GC microelectrodes are reported in Table 3. As expected from the electrochemical results, the GC electrodes were able to record high-quality SEPs with greater SNR as compared to the Pt electrodes, due to their lower impedance. Additionally, the combination of GC and PEDOT-PSS had the highest SNR, again indicating that GC is the superior substrate for the conductive polymer.

We verified the stability and durability of the PEDOT-PSS coatings *in vivo* by measuring the impedance before and after the recording sessions. Comparisons (mean and standard deviation from 6 recording sites each) of *in vivo* impedance spectra of 300 μm Ø PEDOT-PSS-coated GC vs. GC microelectrodes are reported in Supplementary Fig. S4. The *in vivo* impedances were analyzed through electrochemical impedance spectroscopy (EIS) using the two-electrode configuration, as described in Supplementary Information. Representative impedance spectra of PEDOT-PSS-coated Pt microelectrodes before and after recording sessions in rat brain are reported in Supplementary Fig. S4, and show no change before and after the implantation. This result further supports previous evidence on the suitability of PEDOT:PSS for neural interfaces^37^,^38^,^39^,^40^.

### Biocompatibility of microelectrodes

**Figure 7:**
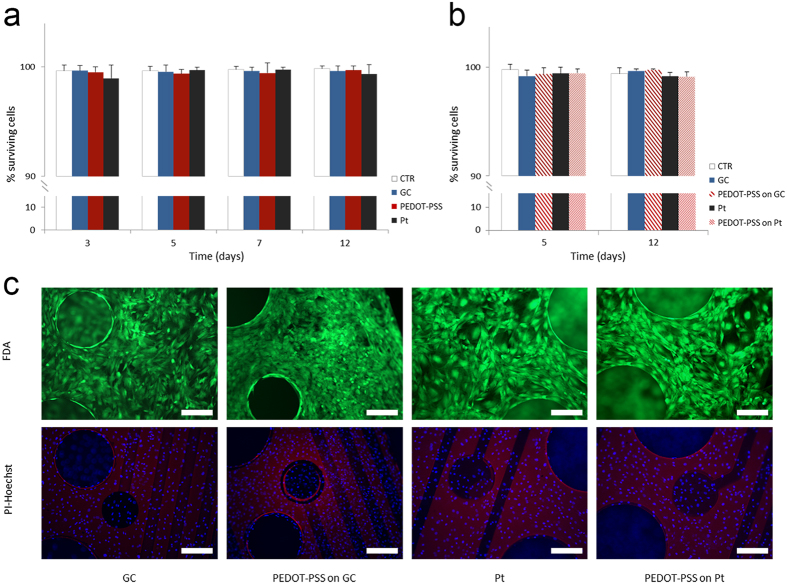
Electrode biocompatibility tests. (**a**) Quantitative analysis of the viability of fibroblasts cells cultured on glass coverslip as control sample (CTR, white bars), glassy carbon (GC, blue bars), PEDOT-PSS (red bars) and Pt (Pt, black bars) substrates at 3, 5, 7, and 12 days *in vitro*. (**b**) Quantitative analysis of the viability of fibroblasts cells cultured on glass coverslip as control sample (CTR, white bars), glassy carbon (GC, blue bars), PEDOT-PSS on GC (thick diagonal red lines), Pt (Pt, black bars) and PEDOT-PSS on Pt (thin diagonal red lines) devices at 5 and 12 days *in vitro*. In a and b the percentages of surviving cells (means ± SD) were calculated based on the ratio of total (Hoechst-positive) nuclei minus dead cells (PI-positive) nuclei divided by the total nuclei. (**c**) Representative images of fibroblasts cells grown on the four different type of devices at day 5 stained with FDA (green-viable cells marker), Hoechst-33342 (blue-total cells nuclei marker) and PI (red-dead cells nuclei marker). Scale bar: 200 μm.

Lastly, in order to study the biocompatibility of the different materials, the cell viability assay was initially performed at different time points (3, 5, 7 and 12 days) on glassy carbon, Pt and PEDOT-PSS ad-hoc produced large substrates by monitoring the cell (fibroblasts) survival. In all cases, the cells were able to adhere to the substrates and to form a thick layer of living cells. The assay demonstrated that cell viability on GC, Pt and PEDOT-PSS substrates was comparable to the control sample (glass coverslip) without any significant difference, in good accord with literature^37^,^54^,^55^,^56^,^57^,^58^,^59^,^60^,^61^,^62^,^63^,^64^,^65^. The percentage of surviving cells progressively increased after 3 days of culture and remained constant for 12 days (Fig. 7). Thereafter the same assay was performed on GC, GC-PEDOT-PSS, Pt and Pt-PEDOT-PSS microelectrode contacts to evaluate the viability response after the manufacturing processes of the neural probes. This test allowed us to test the biocompatibility of the entire structure including the polyimide portion that indeed represents a large part of the array. Fig. 7b shows that even for the whole devices the rate of surviving cells remained very high during the *in vitro* culture, with dense network of cells covering the entire device (Fig. 7c).

## Conclusions

In this paper, we present the superior recording and stimulating capabilities of a new neural electrode material, glassy carbon, with respect to traditional noble metal (Pt) electrodes integrated into thin-film devices. We also demonstrate that GC has a very homogenous surface with a mean roughness of 3.3 nm whereas Pt has a mean roughness of an order of magnitude higher than this. Further, through extensive *in vitro* electrochemical experiments, we demonstrated that glassy carbon electrodes have superior electrical (lower impedance) and electrochemical properties than devices having Pt electrodes of the same diameter, making them an attractive choice for neural applications. The main advantage GC-based devices have over traditional Pt electrodes is the larger EW and electrochemical stability that allows for greater voltage excursions during stimulation protocols. Additionally, when high-resolution applications are necessary, we have demonstrated that conductive polymers such as PEDOT-PSS adhere well to GC and PEDOT-PSS coated GC electrodes have greater CTC than similarly coated metal electrodes. Due to the combination of a very stable GC substrate and a stable, high surface area PEDOT-PSS coating, we have demonstrated the ability to fabricate miniaturized microelectrodes that maintain excellent electrochemical performance and are capable of injecting a current sufficient to evoke sensations in humans. These stable, low-impedance miniaturized microelectrodes open the possibility of improving sensitivity, spatial selectivity and spatial resolution of micro-ECoG array technologies for both recording and stimulation applications.

The electrochemical impedance analysis supports the theory of GC being a better substrate for PEDOT-PSS than Pt, as the PEDOT-PSS-coated GC electrodes exhibit a lower impedance (due to higher capacitance) and better stability than the Pt counterparts. SEM analysis shows a difference in the morphology of PEDOT-PSS deposited onto the two substrates, confirming the fact that the substrates play a key role on the electrical characteristic of the final products.

*In vivo* tests confirmed what the electrochemical characterization had previously showed: measured impedance values of GC microelectrodes were lower than the Pt ones and they were able to record high-quality neural signals with greater SNR. GC also performed much better than Pt as substrate for PEDOT-PSS and enhanced the electrical and electrochemical properties of the conductive polymer. In fact, PEDOT-PSS-coated GC electrodes recorded neural activity with the highest SNR. We have proven (through both *in vitro* and *in vivo* tests) that the combination of GC and PEDOT-PSS has a synergistic effect that can be very useful for high-resolution applications. Finally, we demonstrated the biocompatibility of glassy carbon based thin film devices through cell viability experiments, which showed no difference in cell viability on the GC devices as compared to the positive control.

## Methods

### Glassy Carbon micro-ECoG array fabrication

The fabrication of the thin-film devices used for this study is described in detail elsewhere^20^. In summary, the glassy carbon electrodes were fabricated using the negative photoresist SU-8, which was patterned and pyrolyzed at 1000 °C in inert atmosphere. Subsequently, a layer of photosensitive polyimide (HD Microsystem) was spun and patterned onto the electrodes, as a substrate for the subsequent layers. Metals (Cr and Au) were then deposited on the substrate to create the conductive traces, and finally a second layer of polyimide was spun on the traces to electrically insulate them.

### PEDOT-PSS electrodeposition

Poly(3,4-ethylenedioxythiophene) - poly(sodium 4-styrenesulfonate (PEDOT-PSS) was electrodeposited from a 0.5 M 3,4-ethylenedioxythiophene (EDOT, Sigma-Aldrich, USA) aqueous solution containing 0.6 wt% of poly(sodium 4-styrenesulfonate)(PSS, Sigma-Aldrich, USA). Electrochemical depositions were carried out by subjecting the GC and the Pt microelectrodes to 50 voltage cycling between −0.3 and 0.9 V maintaining a scan rate of 100 mV/s, through a potentiostat/galvanostat (Reference 600, Gamry Instruments, USA) connected to a three-electrode electrochemical cell with a Pt counter electrode and an Ag/AgCl reference electrode.

### Electrochemical characterization

The electrochemical behaviour of the microelectrodes was studied in a 0.9% NaCl aqueous solution, by cyclic voltammetry (CV) and electrochemical impedance spectroscopy (EIS). During the CV tests, the working electrode potential was swept between 0.6 and −0.8 V with a scan rate of 100 mV/s. EIS measurements were performed by superimposing a sine wave (10 mV RMS amplitude) onto the open circuit potential, in the frequency range from 1 to 10^5^ Hz. Linear sweep voltammetry (LSV) was used for the determination of the water reduction and oxidation overpotentials for the different materials, sweeping the working electrode potential between −1.5 and 2 V, with a scan rate of 50 mV/s. EIS, LSV and CV were carried out using a potentiostat/galvanostat (Reference 600, Gamry Instruments, USA) connected to a three-electrode electrochemical cell with a Pt counter electrode and a Ag|AgCl|KCl(sat.) reference electrode. The software ZSimpWin V 3.2 (EChem Software) was used for equivalent circuit modeling of EIS data.

### Optical and surface characterization of coated microelectrodes

GC, Pt and PEDOT-PSS coated microelectrodes were routinely examined via optical microscopy using a Leica Zoom APO 16 equipped with a Leica DFC290 digital camera (Leica Microsystems, Germany). Morphology of the electrodes was studied through scanning electron microscopy, Quanta 450 (FEI Company, Hillsboro, Oregon, USA), using secondary electron detector and Atomic Force Microscopy (AFM-WORKSHOP TT-AFM, Signal Hill, California, USA).

### Surgical procedure

The experimental procedures were carried out in accordance with the guidelines established by the European Communities Council (Directive 2010/63/EU of September 22nd, 2010) and the protocol was approved by the Italian Ministry of Health, authorization n° 332/2015-PR. The experiments were performed on 3 Long-Evans rats (400–500 gr). The animals were bred in the breeding facility of the University of Ferrara.

Experimental subjects were anesthetized with a mixture of Zoletil (Virbac, France; 30 mg/kg) and Xylazine (Bayer, Germany; 5 mg/kg) administered intraperitoneally (i.p.). For the duration of the whole procedure, the depth of anesthesia was monitored by testing the absence of hind limb withdrawal reflex and was maintained by additional intramuscular (i.m.) doses of anesthetic. Body temperature was kept at 37.5 °C by a thermostatically controlled heating pad and Lacrigel (Farmigea, Italy) was placed on eyes to avoid dryness. The anesthetized animals were placed in a stereotaxic apparatus (David Kopf Instruments, USA) provided with ear and bite bars. Subsequently, a craniotomy of 5 × 6 mm along rostro-caudal and medial-lateral axis between bregma and lambda was made over the right somatosensory cortex. The dura mater was left intact and each 12-channel micro-ECoG array was placed epidurally on the area of the cortex representing the vibrissa (according to Paxinos atlas^66^). A ground needle electrode was attached to a screw inserted in the contralateral parietal bone.

The recorded SEPs from vibrissae cortex were elicited by trigeminal nerve electrical stimulation (see below).

### Electrical stimulation and SEPs recording

To elicit neural response, two intramuscular bipolar needle electrodes were manually inserted into the vibrissae pad to electrically stimulate the trigeminal nerve. 100 positive rectangular pulses of 2 mA magnitude and 0.2 ms duration were generated and delivered every 4 seconds (0.25 Hz)^67^ by a WPI A310 pulse generator through a WPI A360R stimulus isolation unit (World Precision Instruments, USA).

The neural signal from 12-channels micro-arrays were acquired from 8 different positions of the vibrissa region of the S1 using a TDT multi-channel recording system 3 (Tucker Davis Technologies, USA) including ZIF-Clip® headstage with unity (1x) gain, the RZ2 real-time processor and the PZ2-256 battery-powered preamplifier. Data was digitized at a sample rate of 3051.8 samples/s at 24 bit resolution, and transferred from RZ2 to the PC by fast fiber optic connection. Then, the acquired traces were digitally low-pass filtered (Butterworth 2nd order) using MATLAB software (Mathworks, USA) to obtain the local field potential (LFP, <300 Hz) and 102 responses were averaged from 50 ms before and 100 ms after stimuli using EEGLAB MATLAB^68^ toolbox and OriginPro (OriginLab, USA).

### Signal-to-noise ratio and signal power calculation

To calculate the SNR, we estimated the signal power by computing as the integral of the spectral power density (SPD) of the signal (evoked activity) and the noise power computed as the integral of the SPD of the noise (spontaneous activity). The SNR has been defined as the ratio between the signal power and the noise power.

The SPDs were estimated using the Multi-Taper Spectral Estimate available in Neuroexplorer 5 software (Next Technology, Littleton, MA). We considered 10 intervals of 60 ms selected from the overall recorded traces (3051.8 samples/s, 3–1000 Hz) and the periodograms (specifically 5 tapers) were calculated for each segment. Regarding the signal, the 10 intervals were set from 5 to 65 ms after the trigger (including the evoked activity), while the 10 intervals of 60 ms of spontaneous activity between stimuli were considered as noise. By this way, each taper was calculated by applying a specially designed windowing function (Slepian function) and all the tapers for a given segment were then averaged to form the periodogram of the segment. This method attempts to reduce the variance of spectral estimates by using this small set of tapers. A set of independent estimates of the power spectrum was computed by pre-multiplying the data by orthogonal tapers which are constructed to minimize the spectral leakage due to the finite length of the data set^69^,^70^. Data are presented as mean ± standard deviation of the SNR calculated for all the recording channels in the 8 different positions.

### Cell viability

Fibroblasts were obtained from culture of rat tail specimens. Briefly, tail biopsies (∼1 cm in length) were obtained from the latter half of the intact tail after skin sterilization with 70% ethanol. Biopsies were further washed in PBS 1X (Thermo Fisher Scientific, US) and cut into small squares (2 × 2 mm approx.) under sterile conditions. 5 to 10 skin pieces were placed in the centre of a 6-well plate covered by a glass coverslip and few drops of ice-cold (4 °C) complete growth medium (60 ml Fetal Bovine Serum (Sigma-Aldrich, Italy) and 500 ml Advanced DMEM, 5 ml 1 m HEPES, 5 ml L-glutamine, 5 ml penicillin/streptomycin (all from Thermo Fisher Scientific, USA)) were added into the space below the coverslip. The medium was changed every 3 days. Upon confluence, the coverslips were removed and the cells were detached from the plate using trypsin (5 min at room temperature). Trypsin was inactivated by adding the same amount of ice-cold complete growth medium while cells were harvested by gently pipetting. Then the suspension was centrifuged (900 rpm, 10 min at 4 °C) and finally the cells were used for biocompatibility assay.

Approximately 85000/cm^2^ fibroblasts were plated over Pt, GC, Poly(3,4 ethylenedioxythiophene) - poly(styrenesulfonate) (PEDOT-PSS)-coated substrates and glass coverslips as control sample (CTR). Likewise, fibroblasts were seeded over Pt, PEDOT-PSS on Pt, GC and PEDOT-PSS on GC microelectrodes devices. After the cells attached to the surface, the cell viability was analyzed at 3, 5, 7 and 12 days for the substrates or at 5 and 12 days for the devices. Viability was evaluated by rating the surviving cells over the total number of cells. Samples were incubated for 3 min at room temperature in Ringer-Locke solution containing propidium iodide (PI; Sigma-Aldrich, Italy; 15 μg/ml), fluorescein diacetate (FDA; Sigma-Aldrich, Italy; 5 mg/ml) and Hoechst-33342 (Sigma-Aldrich, Italy; 3.3 μg/ml). After incubation, samples were washed once in Ringer-Locke solution and immediately imaged. At least 10 different fields of view were acquired for each sample. Images were acquired and analyzed using an Olympus BX51 fluorescence microscope (Olympus, USA) equipped with X-Cite 120 fluorescence illumination system (EXFO, Canada), with a color CX-9000 digital camera (MicroBrightField, USA) coupled with the NeuroLucida software (MicroBrightField, USA). For each field, the ratio of PI-positive cells over the total number of nuclei, identified by Hoechst-33342, was calculated.

## Additional Information

**How to cite this article**: Vomero, M. *et al*. Highly Stable Glassy Carbon Interfaces for Long-Term Neural Stimulation and Low-Noise Recording of Brain Activity. *Sci. Rep.* **7**, 40332; doi: 10.1038/srep40332 (2017).

**Publisher's note:** Springer Nature remains neutral with regard to jurisdictional claims in published maps and institutional affiliations.

## Supplementary Material

Supplementary Information

## Figures and Tables

**Table 1:** EIS parameters of GC and Pt microelectrodes obtained by fitting the experimental data to the model shown in Fig. 2c,d.

	R_S_ (W)	Q_o_ (S s^n^)	n	R_CT_ (W)	W(Y_0_) (S s^0.5^)
GC	1271	6.7E-8	0.93	2.9E7	1.9E-5
Pt	1219	2.8E-8	0.87	1.99E6	1.5E-8

See text for abbreviations.

**Table 2:** Representative EIS parameters of PEDOT-PSS/GC and PEDOT-PSS/Pt microelectrodes obtained by fitting the experimental data to the model shown in Fig. 4c,d.

	R_D_ (Ω)	C_EIS_ (mF)	C_CV_ (μF)
**PEDOT/GC**	327	39	31
**PEDOT/Pt**	426	22	13

See text for abbreviations.

**Table 3:** SNR in low frequency band (0–250 Hz) of Pt, GC, PEDOT-PSS coated Pt and PEDOT-PSS coated GC coated microelectrodes.

SNR			
Pt	GC	PEDOT-PSS/Pt	PEDOT-PSS/GC
8.27 ± 10.59	16.44 ± 12.67	13.44 ± 11.00	16.05 ± 11.61
