# Supplementary material for: Highly Stable Glassy Carbon Interfaces for Long-Term Neural Stimulation and Low-Noise Recording of Brain Activity
Source: Sci Rep. 2017 Jan 13;7:40332. doi: 10.1038/srep40332 (PMC5234039; doi:10.1038/srep40332)
Supplement: Supplementary Information [file srep40332-s1.pdf]

Supplementary Information

Highly Stable Glassy Carbon Interfaces for Long-Term Neural Stimulation and Low-Noise Recording of Brain Activity

Maria Vomero[1,2*], *Elisa Castagnola*[3], *Francesca Ciarpella*[3,4], *Emma Maggiolini*[3], *Noah Goshi*[1,2], *Elena Zucchini*[4], *Stefano Carli*[3], *Luciano Fadiga*[3,4], *Sam Kassegne*[1,2], *Davide Ricci*[3]

[1] MEMS Research Lab., Department of Mechanical Engineering, College of Engineering, San Diego State University, 5500 Campanile Drive, PS127 San Diego, CA 92182-1323, USA

[2] Center for Sensorimotor Neural Engineering (CNSE)

[3] Center for Translational Neurophysiology of Speech and Communication, Istituto Italiano di Tecnologia, Via Fossato di Mortara 17/19, 44121, Ferrara, Italy

[4] Section of Human Physiology, University of Ferrara, Via Fossato di Mortara 17/19, 44121, Ferrara, Italy

*address correspondences to vomero@rohan.sdsu.edu

	Impedance Magnitude [kΩ]			CTC [mC/cm²]	EW [V]
	10 Hz	*100 Hz*	*1 kHz*	*EW range*	
**300µm Ø Pt**	564.5±311.3	89.6±42.2	14.3±6.9	8.6±3.5	1.4
**300µm Ø GC**	386.1±99.1	61.7±23.8	11.0±5.4	14.3±3.8	2.4
**300µm Ø PEDOT-PSS/GC**	1.5±0.5	1.0±0.1	0.9±0.1	125.7± 41.6	1.4
**300µm Ø PEDOT-PSS/Pt**	1.9±0.3	1.3±0.1	1.2±0.1	71.0±11.5	1.4
**60µm Ø GC**	1.1E4±6.4E3	2.8E3±1.4E3	417.5±105.7	46.9±8.9	2.4
**60µm Ø PEDOT-PSS/GC**	12.4±2.4	4.8±0.7	3.6±0.4	893.5±137.8	1.4

Supplementary Table S1. Comparison of electrochemical properties of GC, Pt and PEDOT-PSS/GC and PEDOT-PSS/Pt electrodes. Impedance at 10 Hz, 100 Hz, 1 kHz, Charge Transfer Capacity (CTC) and Electrochemical Window (EW) of the Pt, Glassy Carbon (GC) and PEDOT-PSS-coated Pt and GC microelectrodes.

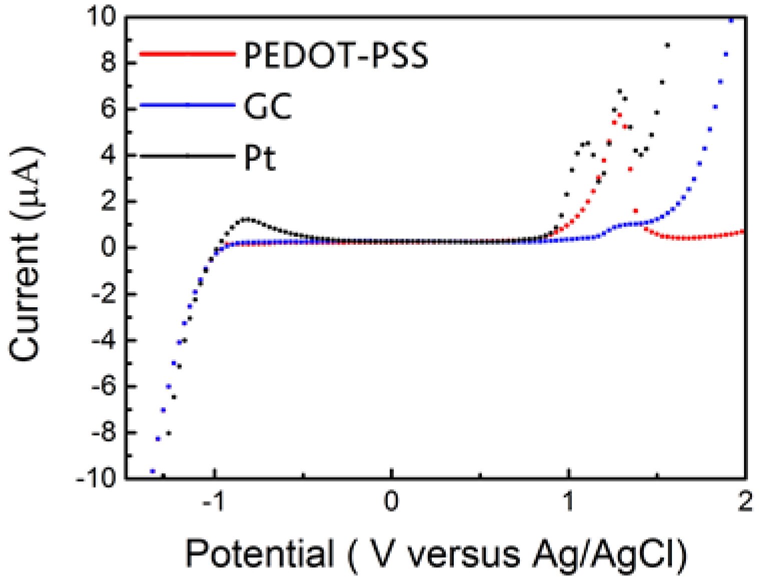

Supplementary Figure S1. Linear sweep voltammetry of Pt, GC, PEDOT-PSS-coated microelectrodes. (Between 2 and −1.5 V vs. Ag/AgCl, scan rate of 50 mV/s).

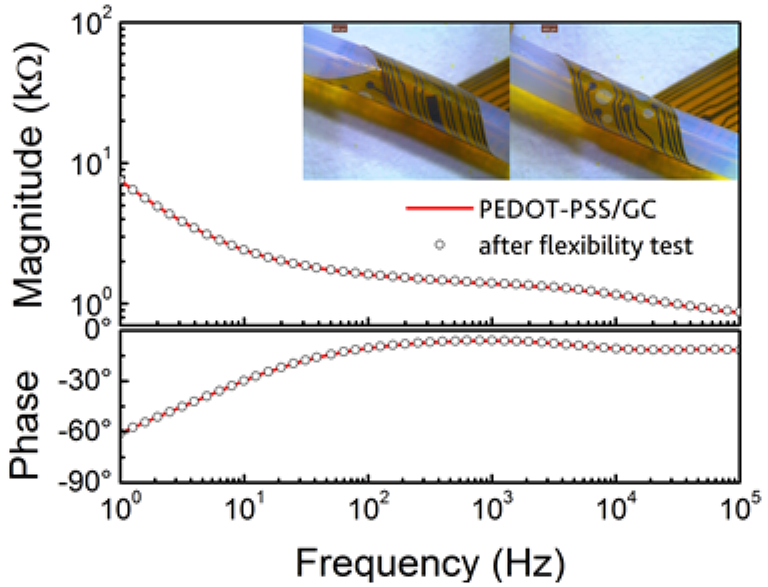

Supplementary Figure S2. Flexibility tests. Representative impedance spectra of a 300µm Ø PEDOT-PSS coated GC microelectrodes before and after flexibility tests. Optical pictures of the flexible device rolled around a 1.25 mm Ø tube.

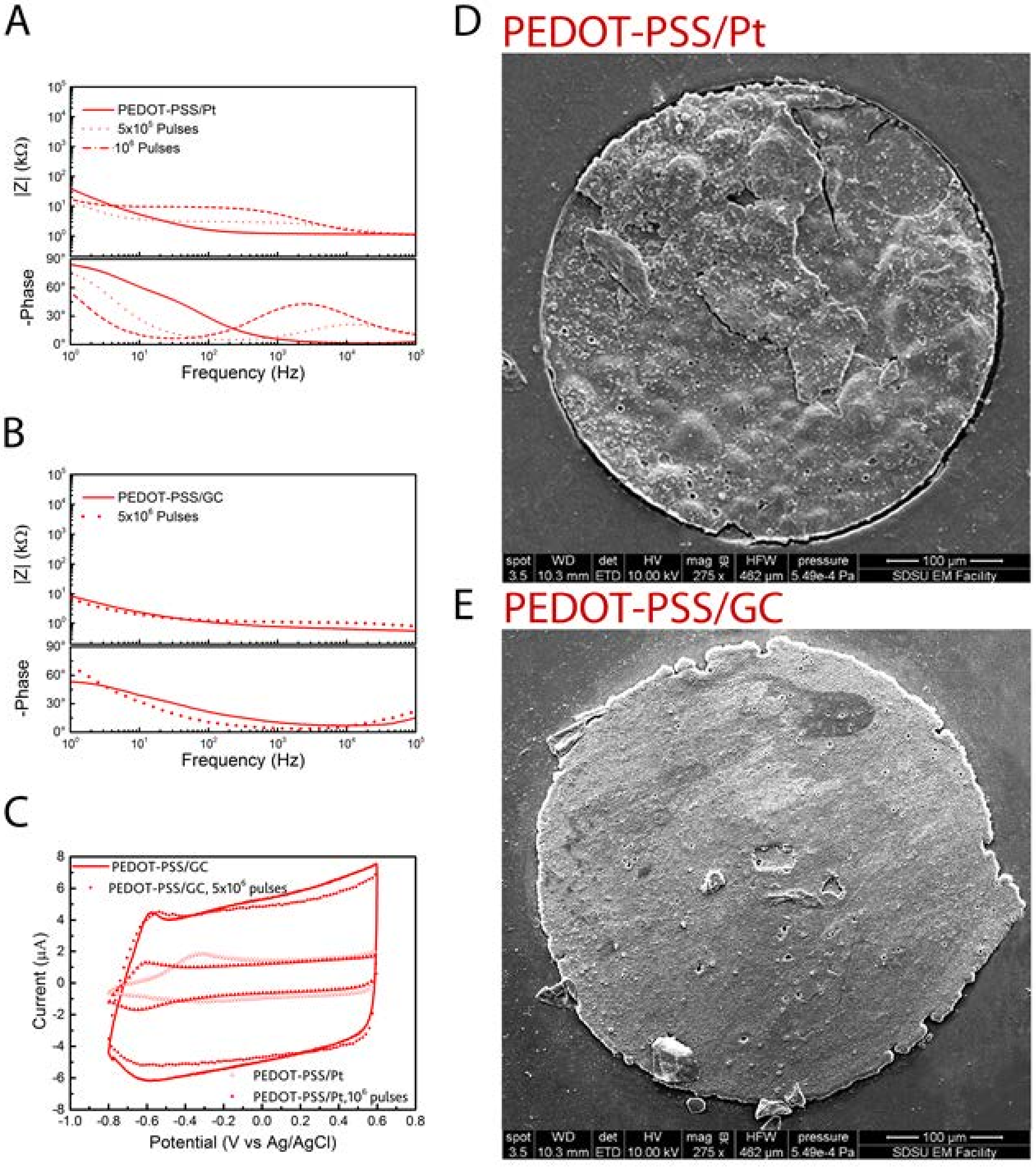

Supplementary Figure S3. Effects of stimulation pulsing on PEDOT-PSS/GC vs PEDOT-PSS/Pt. Representative impedance spectra before and after stimulation experiments of PEDOT-PSS coating on GC (A) and PEDOT-PSS coating on Pt (B) 300µm Ø microelectrodes. (C) Representative cyclic voltammograms of PEDOT-PSS coating on GC and PEDOT-PSS coating on Pt, before and after stimulation experiments. (D, E) Representative SEM images of PEDOT-PSS-coated Pt (D) and GC (E) 300µm Ø microelectrodes, respectively after 1 (Pt) and 5 (GC) million pulses.

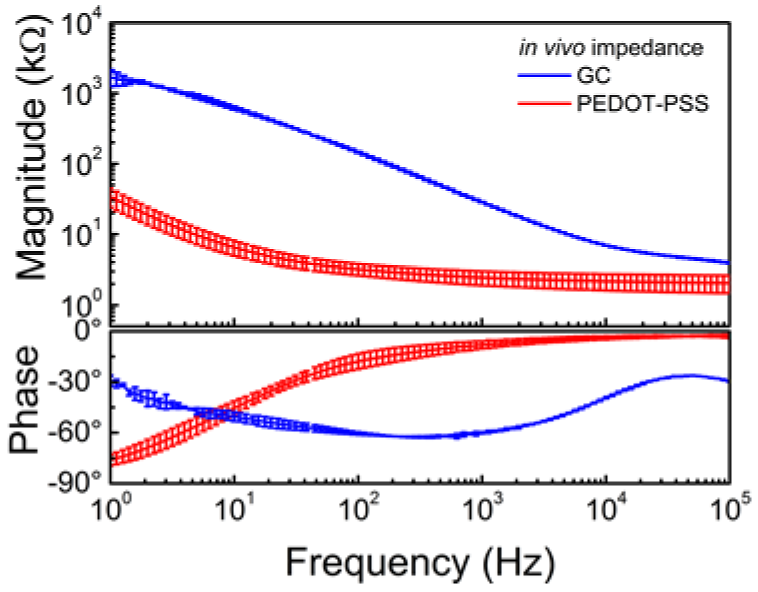

Supplementary Figure S4. ***In-vivo* impedance results.** Comparison (mean and standard deviation from 6 recording sites each) of *in vivo* impedance spectra of 300µm Ø PEDOT-PSS-coated GC versus GC microelectrodes.

***In-vivo* impedance measurements.**
The *in vivo* impedances were analyzed through electrochemical impedance spectroscopy (EIS) using the two-electrode configuration. The 12 microelectrodes of each devices were referenced to a low impedance stainless steel bone screw inserted into the skull. The two-electrode method is suitable for measuring impedance from microelectrodes due to the large difference in impedance relative to the reference and the small current that passes through the circuit. During the EIS measurements, a sine wave (10 mV RMS amplitude) was imposed onto the open circuit potential while varying the frequency from 1 to 10^5 Hz. EIS were carried out using a potentiostat/galvanostat (Reference 600, Gamry Instruments, USA).

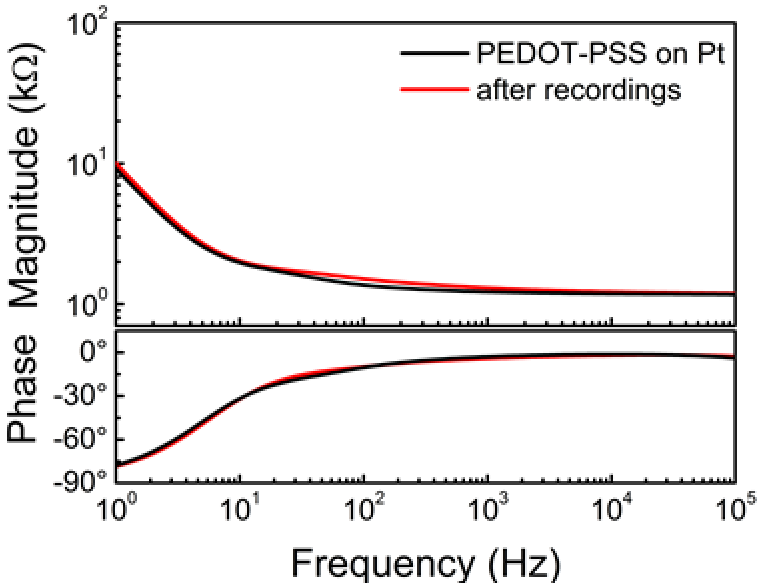

Supplementary Figure S5. Representative impedance spectra of a PEDOT-PSS-coated Pt microelectrode before and after recording sessions in rat brain.

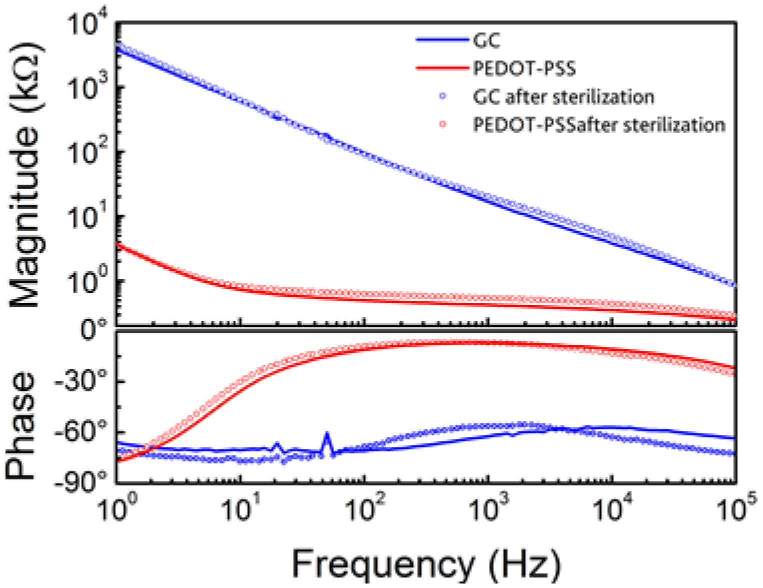

Supplementary Figure S6. Representative Impedance spectra of a GC and a PEDOT-PSS-coated CG microelectrodes before and after sterilization tests.
